# Pretreatment with a GnRH agonist and hormone replacement treatment protocol could not improve live birth rate for PCOS women undergoing frozen-thawed embryo transfer cycles

**DOI:** 10.1186/s12884-021-04293-4

**Published:** 2021-12-18

**Authors:** Xitong Liu, Juanzi Shi, Haiyan Bai, Wen Wen

**Affiliations:** The Assisted Reproduction Center, Northwest Women’s and Children’s Hospital, Xi’an, China

**Keywords:** Frozen-thawed embryo transfer, Endometrial preparation, Live birth rate, Polycystic ovary syndrome

## Abstract

**Background:**

The ideal protocols of endometrial preparation for polycystic ovary syndrome (PCOS) patients are lacking and need further declaration. Our objective was to compare the clinical outcomes of frozen-thawed embryo transfer (FET) with and without pretreatment gonadotropin-releasing hormone agonist (GnRHa) in PCOS patients.

**Methods:**

In this retrospective cohort study, we used propensity score matching (PSM) to compare the live birth rate between patients who underwent FET with hormone replacement treatment (HRT) and patients with GnRHa pretreatment (GnRHa + HRT). Patients using GnRHa + HRT (*n* = 514) were matched with 514 patients using HRT.

**Results:**

The live birth rate was higher in the GnRHa + HRT group compared with the HRT group with no significant difference (60.12% vs 56.03%, *p* = 0.073). The clinical pregnancy rate (75.29% vs 70.62%), miscarriage rate (14.20% vs 13.81%) and ectopic pregnancy rate (0.39% vs 0.19%) were similar between the two groups. The preterm birth rate in GnRHa + HRT was higher than HRT (20.23% vs 13.04%). No difference was found in live birth between GnRHa +HRT and HRT before adjusting for covariates (crude OR 1.22, 95%CI, 0.99–1.51, *p* = 0.062) and after PSM (OR 1.47, 95%CI, 0.99–2.83, *p* = 0.068). In addition, there is a marginally difference after adjusting for covariates (aOR 1.56, 95%CI, 1.001–2.41, *p* = 0.048), this finding with *p*-value close to 0.05 represent insufficient empirical evidence. Similar results were obtained after propensity score matching in the entire cohort.

**Conclusions:**

GnRHa pretreatment could not improve the live birth rate in women with PCOS.

## Background

Polycystic ovary syndrome (PCOS) is a common endocrine syndrome and affects between 8 and 18% of women [1]. It is characterized by a combination of the following three criteria: oligoovulation or anovulation, polycystic ovaries, and hyperandrogenism [2]. This syndrome results in luteinizing hormone (LH) and follicle-stimulating hormone (FSH) ratio imbalance, cardiovascular diseases, obesity, infertility, and other health issues.

In recent years, the freeze-all policy of cryopreserving all embryos produced in an IVF cycle to transfer later has become more appropriate when there is a risk of ovarian hyperstimulation syndrome (OHSS), high progesterone level, preimplantation genetic testing (PGT), abnormal endometrium, and other conditions. Studies have demonstrated comparable outcomes between fresh and frozen-thawed embryo transfer (FET) [3–5]. As national consensus continues to endorse single embryo transfer [6, 7], more supernumerary embryos are available for cryopreservation. Patients with PCOS have hyper-responsiveness to gonadotropin stimulation. Women with PCOS tend to have more oocytes retrieved and an elevated estradiol level. The altered hormone level may adversely affect endometrial receptivity in fresh embryo transfer. FET has been widely used for PCOS patients to minimize the risk of OHSS. Protocols described in FET include natural cycle, hormone replacement treatment (HRT) cycle, and down-regulation with GnRH agonists (GnRHa). Thus, HRT with and without GnRHa becomes the most common method of endometrial preparation for PCOS patients. HRT regimens avoid the cost of GnRHa and may have similar clinical outcomes. However, the GnRHa cycle offers the most control over the timing of the cycle and minimizes cancellation rates. The GnRHa cycles also eliminate the possible detrimental role of high LH on the oocyte quality and implantation rate.

Currently, the optimal means for endometrial preparation for PCOS patients is a topic of ongoing controversy [8, 9]. Most studies focused on ovulatory women instead of PCOS patients, and existing studies have been conflicting. The Cochrane review showed there is insufficient evidence on the use of any particular intervention for endometrial preparation in women undergoing FET [10]. Therefore, we aimed to evaluate the live birth rate between two different endometrial preparations (HRT and GnRHa + HRT) for PCOS patients.

## Methods

### Sample size estimation

According to the statistical data of our center, the live birth rate of HRT was 0.65. Assuming α = 0.05 and 80% power, it was calculated that 387 frozen–thawed embryos were required for transfer in each group to demonstrate a difference of 10% in live birth rate.

### Study design and patients

**Fig. 1:**
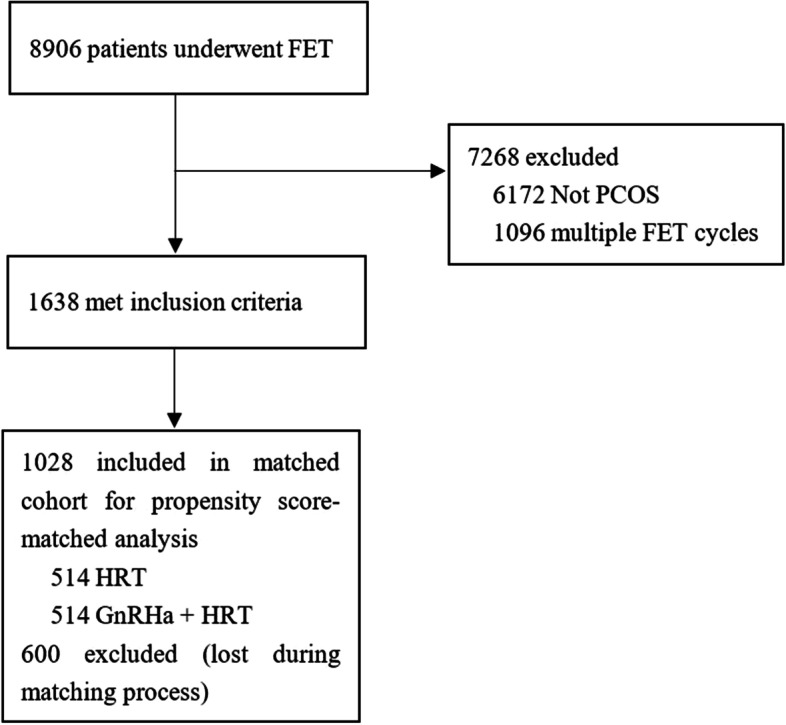
Selection of study population

This retrospective cohort study considered 8906 consecutive patients undergoing FET between June 2014 and December 2017 in the Center for Assisted Reproductive Technology of Northwest Women’s and Children’s Hospital, China. The selection of the study population was shown in Fig. 1. Women who underwent the first cycle of FET were considered eligible for the study if they were diagnosed with PCOS according to Rotterdam criteria and had previous in vitro fertilization cycles with embryo cryopreservation, regardless of age, diagnosis, embryo stage, or the number of transferred embryos. We excluded patients who were not diagnosed with PCOS and multiple FET cycles. We excluded 7268 patients who were not PCOS or multiple FET cycles. We used propensity score matching (PSM) to compare the live birth rate between patients who underwent FET with hormone replacement treatment (HRT) and patients with GnRHa pretreatment (GnRHa + HRT). Patients using GnRHa + HRT (*n* = 514) were matched with 514 patients using HRT.

### Endometrial preparation protocols

#### Hormone replacement treatment (HRT) cycle

Supplementation with oral estrogens (4-6 mg/day; Progynova; Bayer Schering Pharma AG, Berlin, Germany) was administered on day 2–5 of the menstrual cycle. Serum progesterone level and ultrasound were performed 10–12 days later. If endometrial thickness > 7 mm and serum progesterone level < 1.5 ng/ml, vaginal progesterone was administered to achieve endometrial transformation, and FET was scheduled. Embryo transfer was performed after 3 days (cleavage stage embryos) or 5 days (blastocyst stage embryos).

#### GnRHa + HRT cycle

GnRHa (3.75 mg; Diphereline, Ipsen Pty Ltd., France) was injected on day 2–5 of menstruation. Oral estrogens were administered 30 days after GnRHa and HRT protocol was then performed as previously described.

### Luteal phase support and pregnancy confirmation

Six hundred mg of vaginal micronized progesterone (Utrogestan, Besins, Belgium), 30 mg oral progesterone (Dupbaston, Abbott Biologicals B.V., Netherlands) daily, divided into three dosages, and estrogens (the same dosage as endometrial preparation) were administrated from the day of embryo transfer. Serum β-hCG levels were measured 14 days after embryo transfer. If serum β-hCG was positive, luteal support continued and an ultrasound was carried out 4–5 weeks later.

### Definition of clinical outcomes

The definition of clinical outcomes was confirmed according to the ASRM consensus published in the year of 2017 [11]. Clinical pregnancy was defined as an intrauterine gestational sac at 7 weeks gestation. Preterm birth was defined as a birth that occurs before 37 weeks of gestational age. Ectopic pregnancy was defined as a pregnancy that occurs outside of the uterine cavity. Miscarriage was defined as a pregnancy loss before 24 weeks of pregnancy. Live birth was defined as the delivery of any viable infant ≥24 weeks of gestation. All the patients were followed up until 1 year after embryo transfer.

### Statistical analysis

Data are presented as mean ± standard deviation for continuous variables. Counts and proportions were used for the categorical variables [n (%)]. One-way analysis of variance was used for continuous variables, and the Pearson’sχ^2^ test was used for categorical variables with Fisher’s exact test when necessary. Logistic regression, presented as unadjusted odds ratio (crude odds ratio (OR)) or adjusted odds ratio (aOR) with 95% confidence interval (CI), was performed. A variance inflation factor (VIF) was calculated to test for collinearity among the predictors. We adjusted for covariates that, when added to this model, changed the matched odds ratio by at least 10% in the multivariable regression analyses. In the adjusted model: we adjusted female age at embryo transfer, female age at oocyte retrieval, infertility duration, infertility type, cause of infertility, antral follicle count (AFC), body mass index (BMI), endometrial thickness, duration of endometrial preparation, triple-line endometrial pattern, protocol in the fresh cycle, gonadotropin duration, gonadotropin dosage, no. of oocyte retrieved, no. of transferred embryos, no. of available embryos, no. of good quality embryos transferred, embryo stage and fertilization type. Data were analyzed using the statistical packages R (The R Foundation; http://www.r-project.org;version 3.4.3) and Empower (R) (www.empowerstats.com, X&Y solutions, inc. Boston, Massachusetts). The level of significance was set at *p* < 0.05.

### Propensity score matching (PSM)

**Fig. 2:**
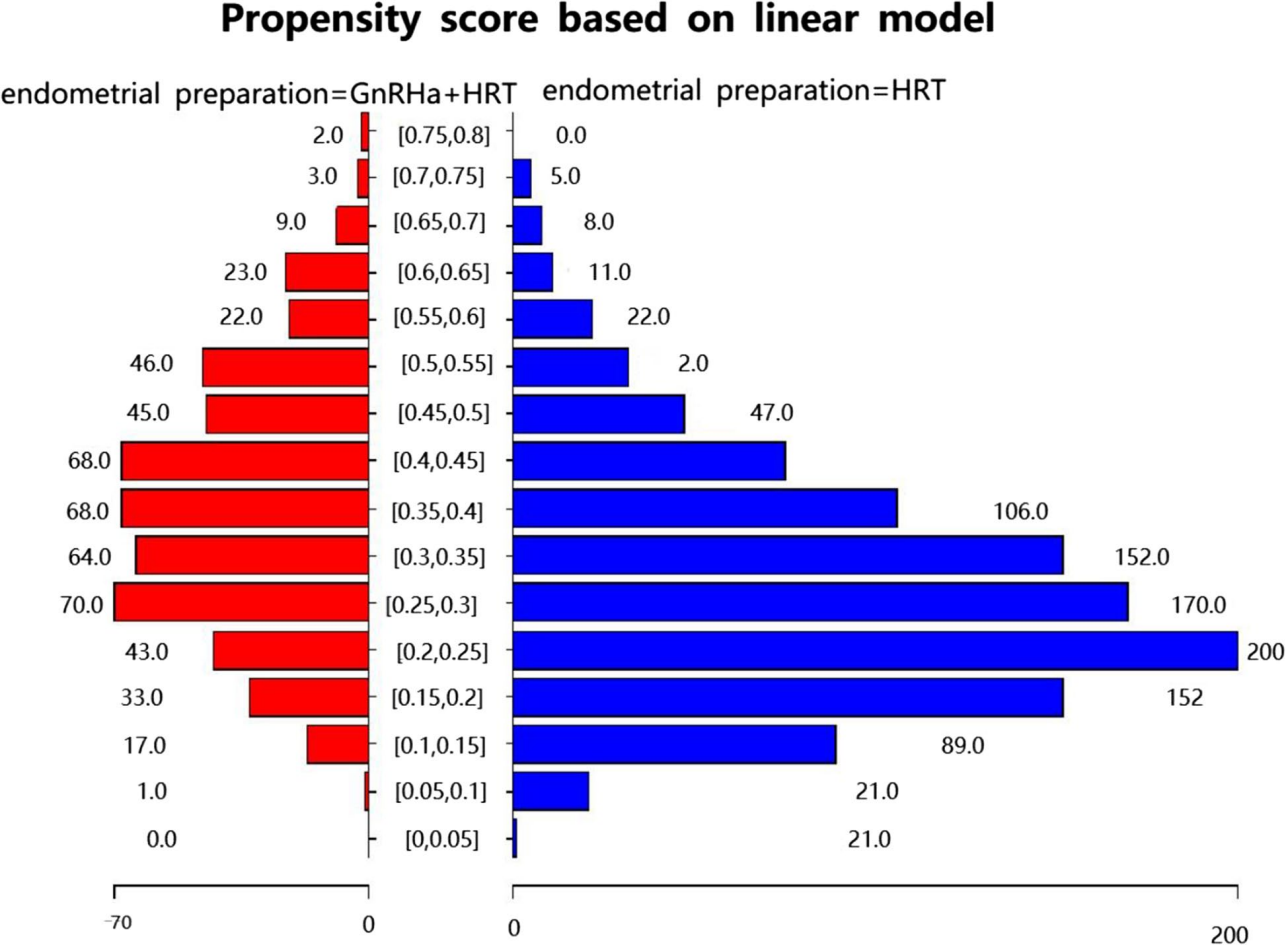
Propensity score in two groups

Given the differences in the baseline characteristics between the two groups, propensity-score matching was used to identify a cohort of women with similar baseline characteristics. A propensity score for endometrial preparation was estimated applying a multivariable logistic regression model, with 8 covariates, including female age at embryo transfer, BMI, the number of oocytes retrieved, endometrial thickness on the day of progesterone administration, triple-line endometrial pattern, embryo stage, the number of available embryos, and the number of good quality embryos transferred as covariates. Matching was performed with the use of a 1:1 matching protocol without replacement, with a caliper width equal to 0.05 of the standard deviation of the logit of the propensity score. Propensity score in two groups was shown in Fig. 2.

## Results

A total of 1638 PCOS cycles of frozen-thawed embryo transfers were performed: 1115 cycles of HRT and 523 cycles of GnRHa + HRT. In the propensity score-matched cohort, 514 patients using GnRHa + HRT were matched with 514 patients using HRT. Variable distribution after PSM was shown in Table 1. The patients’ baseline characteristics were described in Table 2. After matching, the baseline characteristics of the patients were similar in the two groups. The number of oocytes retrieved was lower in the GnRHa + HRT group than HRT (17.71 ± 7.52 vs 19.15 ± 8.41, *p* = 0.004). Gonadotropin duration (12.73 ± 8.29 vs 11.23 ± 3.18, *p* < 0.001) in fresh cycles were higher in GnRHa + HRT group than HRT. Other demographic characteristic was similar between the two groups.

**Table 1:** Variable distribution after PSM

Variables	HRT	GnRHa+HRT	*P* value
Female age at embryo transfer	(514) 28.96 ± 3.48	(514) 29.08 ± 3.18	0.55
BMI	(514) 24.15 ± 3.60	(514) 24.15 ± 3.73	0.98
Number of oocytes retrieved	(514) 19.15 ± 8.41	(514) 17.71 ± 7.52	0.01
EM thickness	(514) 10.00 ± 1.62	(514) 10.18 ± 1.55	0.06
Number of transferrable embryos	(514) 9.68 ± 5.63	(514) 8.70 ± 4.68	0.01
Number of top-quality embryos	(514) 1.31 ± 2.92	(514) 1.99 ± 3.20	< 0.01
Triple-line endometrial pattern			0.14
A	55 (10.7)	74 (14.4)	
B	429 (83.5)	405 (78.8)	
C	30 (5.8)	35 (6.8)	
Type of embryo transferred			0.35
Cleavage stage	157 (30.5)	172 (33.5)	
Blastocyst stage	357 (69.5)	342 (66.5)

**Table 2:** Baseline characteristics between different endometrial preparation groups

	Matched Cohort (*n* = 1028) ^a^			Entire Cohort (*n* = 1638) ^b^		
Variable	HRT (n = 514)	GnRHa+HRT (n = 514)	*P*-value^¶^	HRT (*n* = 1115)	GnRHa+HRT (*n* = 523)	*P*-value^¶^
Female age at oocyte retrieval (years)	28.53 ± 3.44	28.53 ± 3.16	0.698	28.16 ± 3.35	28.49 ± 3.17	0.059
Female age at embryo transfer (years)	28.96 ± 3.48	29.08 ± 3.18	0.330	28.61 ± 3.38	29.08 ± 3.16	0.007
Infertility duration (years)	3.59 ± 2.31	3.60 ± 2.12	0.500	3.59 ± 2.29	3.61 ± 2.13	0.896
BMI (kg/m^2^)	24.15 ± 3.60	24.15 ± 3.73	0.886	23.96 ± 3.49	24.12 ± 3.74	0.396
AFC (n)	22.22 ± 3.28	22.51 ± 3.33	0.197	22.30 ± 3.36	22.49 ± 3.35	0.285
Number of oocytes retrieved (n)	19.15 ± 8.41	17.71 ± 7.52	0.004	19.95 ± 8.30	17.65 ± 7.54	< 0.001
Fertilization type (n, %)			0.769^‡^			0.871^‡^
IVF	407 (79.18%)	413 (80.35%)		904 (81.08%)	421 (80.50%)	
ICSI	88 (17.12%)	80 (15.56%)		172 (15.43%)	81 (15.49%)	
IVF + ICSI	19 (3.70%)	21 (4.09%)		39 (3.50%)	21 (4.02%)	
Infertility type (n, %)			0.690^‡^			0.647^‡^
Primary infertility	372 (72.66%)	367 (71.54%)		783 (70.35%)	373 (71.46%)	
Secondary infertility	140 (27.34%)	146 (28.46%)		330 (29.65%)	149 (28.54%)	
Cause of infertility (n, %)			0.431^‡^			0.371^‡^
Tubal	31 (6.04%)	20 (3.89%)		69 (6.19%)	22 (4.21%)	
Ovulation disorder	185 (36.06%)	184 (35.80%)		388 (34.83%)	186 (35.56%)	
Male factor	12 (2.34%)	11 (2.14%)		29 (2.60%)	11 (2.10%)	
Mixed factor	285 (55.56%)	299 (58.17%)		628 (56.37%)	304 (58.13%)	
Protocol in the fresh cycle (n, %)			0.159^‡^			0.477^‡^
Agonist	460 (89.49%)	474 (92.22%)		1007 (90.31%)	482 (92.16%)	
Antagonist	53 (10.31%)	40 (7.78%)		105 (9.42%)	40 (7.65%)	
Other	1 (0.19%)	0 (0.00%)		3 (0.27%)	1 (0.19%)	
Gonadotropin dosage (IU)	2081.08 ± 924.44	2152.95 ± 965.73	0.223	2001.76 ± 891.03	2135.55 ± 979.92	0.006
Gonadotropin duration (days)	11.23 ± 3.18	12.73 ± 8.29	< 0.001	10.89 ± 3.12	12.62 ± 8.31	< 0.001

^‡^*χ*2 test, ^¶^Student’s *t* test

^a^ Propensity score-matched cohort, ^b^ before propensity score matching

Outcomes of two groups for endometrial preparation were shown in Table 3. The duration of endometrial preparation was longer in the GnRHa + HRT group (11.62 ± 3.54 vs 11.00 ± 3.74, *p* = 0.010). The endometrial thickness on the day of P administration was thicker in the GnRHa + HRT group (10.18 ± 1.55 vs 10.00 ± 1.62, *p* = 0.030). No. of the available embryos in the GnRHa + HRT group were lesser than HRT (8.70 ± 4.68 vs 9.68 ± 5.63, *p* = 0.003). The preterm birth rate was higher in GnRHa + HRT than HRT (20.23% vs 13.04%, *p* = 0.002). Clinical pregnancy rate, miscarriage rate, ectopic pregnancy rate, the live birth rate was not statistically different between the two groups before and after PSM.

**Table 3:** Outcomes of two groups for endometrial preparation

	Matched Cohort (*n* = 1038) ^a^			Entire Cohort (n = 1638) ^b^		
Variable	HRT (n = 514)	GnRHa+HRT (n = 514)	*P*-value^¶^	HRT (n = 1115)	GnRHa+HRT (n = 523)	*P*-value^¶^
Duration of endometrial preparation (days)	11.00 ± 3.74	11.62 ± 3.54	0.010	11.35 ± 3.77	11.61 ± 3.58	0.470
Endometrial thickness on the day of progesterone administration (mm)	10.00 ± 1.62	10.18 ± 1.55	0.030	9.76 ± 1.54	10.18 ± 1.55	< 0.001
Triple-line endometrial pattern (%)			0.144^‡^			0.008^‡^
A	55 (10.70%)	74 (14.40%		103 (9.24%)	75 (14.34%)	
B	429 (83.46%)	405 (78.79%)		931 (83.50%)	413 (78.97%)	
C	30 (5.84%)	35 (6.81%)		81 (7.26%)	35 (6.69%)	
Embryo stage (n, %)			0.316^‡^			< 0.001^‡^
Cleavage stage	157 (30.54%)	172 (33.46%)		282 (25.29%)	174 (33.27%)	
Blastocyst stage	357 (69.46%)	342 (66.54%)		833 (74.71%)	349 (66.73%)	
Number of transferred embryos (n, %)	1.75 ± 0.46	1.76 ± 0.45	0.493	1.74 ± 0.46	1.76 ± 0.45	0.428
Number of the available embryos (n, %)	9.68 ± 5.63	8.70 ± 4.68	0.003	10.08 ± 5.26	8.69 ± 4.70	< 0.001
Number of good quality embryos transferred (n, %)	1.14 ± 0.77	1.06 ± 0.78	0.117	1.15 ± 0.75	1.06 ± 0.78	0.025
Clinical pregnancy rate (n, %)	363 (70.62%)	387 (75.29%)	0.092^‡^	811 (72.74%)	394 (75.33%)	0.266^‡^
Preterm birth rate (n, %)	67 (13.04%)	104 (20.23%)	0.002^‡^	157 (14.08%)	105 (20.08%)	0.002^‡^
Miscarriage rate (n, %)	71 (13.81%)	73 (14.20%)	0.857^‡^	159 (14.26%)	73 (13.96%)	0.870^‡^
Ectopic pregnancy rate (n, %)	1 (0.19%)	2 (0.39%)	0.563^‡^	4 (0.36%)	2 (0.38%)	0.941^‡^
Live birth rate (n, %)	288 (56.03%)	309 (60.12%)	0.073^‡^	619 (55.52%)	316 (60.42%)	0.062^‡^
Birth weight (kg)	3.10 ± 0.71	3.06 ± 0.68	0.645	3.07 ± 0.72	3.07 ± 0.68	0.914
Fetal sex (n, %)			0.583^‡^			0.783^‡^
Female	137 (50.18%)	149 (47.91%)		290 (46.85%)	152 (47.80%)	
Male	136 (49.82%)	162 (52.09%)		329 (53.15%)	166 (52.20%)	

^‡^*χ*2 test, ^¶^Student’s *t* test

^a^Propensity score-matched cohort, ^b^ before propensity score matching

Univariate analysis was performed to evaluate each variable’s effect on the live birth rate (Table 4). BMI and AFC were negatively associated with live birth, while endometrial thickness on the day of progesterone administration, no. of the available embryos, number of transferred embryos, number of good quality embryos transferred, and blastocyst stage embryo transfer was positively associated with live birth.

**Table 4:** Univariate analysis for live birth rate in the entire cohort

Covariate	Statistics	OR (95%CI)	*P*-value^¶^
Female age at oocyte retrieval (years)	28.26 ± 3.30	0.997 (0.97, 1.03)	0.859
Female age at embryo transfer (years)	28.76 ± 3.32	0.99 (0.96, 1.02)	0.446
BMI (kg/m^2^)	24.01 ± 3.57	0.93 (0.91, 0.96)	< 0.001
Infertility duration (years)	3.60 ± 2.24	0.97 (0.93, 1.01)	0.154
Infertility type (%)			
primary infertility	70.70	Reference	
secondary infertility	29.30	0.95 (0.76, 1.17)	0.611^‡^
AFC (n)	22.36 ± 3.36	0.97 (0.94, 0.99)	0.020
Endometrial thickness on the day of progesterone administration (mm)	9.90 ± 1.55	1.14 (1.07, 1.22)	< 0.001
Fertilization type (n, %)			
IVF	1325 (80.89%)	Reference	
ICSI	253 (15.45%)	0.77 (0.59, 1.01)	0.057^‡^
IVF + ICSI	60 (3.66%)	0.82 (0.49, 1.38)	0.450^‡^
Protocol in the fresh cycle (n, %)			
Agonist	1489 (90.90%)	Reference	
Antagonist	145 (8.85%)	0.72 (0.51, 1.01)	0.057^‡^
Other	4 (0.24%)	0.24 (0.03, 2.34)	0.220^‡^
Gonadotropin duration (days)	11.44 ± 5.41	1.002 (0.98, 1.02)	0.804
Gonadotropin dosage (IU)	2044.48 ± 922.16	0.9999 (0.9998, 1.0000)	0.147
Number of oocytes retrieved (n)	19.22 ± 8.14	1.01 (1.00, 1.02)	0.151
Number of the available embryos (n)	9.64 ± 5.12	1.03 (1.01, 1.05)	0.001
Number of transferred embryos (n, %)			
1	431 (26.31%)	Reference	
2	1192 (72.77%)	1.46 (1.17, 1.82)	< 0.001^‡^
3	15 (0.92%)	0.86 (0.31, 2.42)	0.779^‡^
Number of good quality embryos transferred (n, %)			
0	381 (23.26%)	Reference	
1	673 (41.09%)	1.69 (1.31, 2.18)	< 0.001^‡^
2	583 (35.59%)	3.03 (2.32, 3.97)	< 0.001^‡^
3	1 (0.06%)	0.00 (0.00, inf.)	0.970^‡^
Embryo stage (n, %)			
Cleavage stage	456 (27.84%)	Reference	
Blastocyst stage	1182 (72.16%)	1.32 (1.06, 1.64)	0.013^‡^
Triple-line endometrial pattern (n, %)			
A	178 (10.87%)	Reference	
B	1344 (82.05%)	0.97 (0.71, 1.33)	0.846^‡^
C	116 (7.08%)	0.66 (0.41, 1.06)	0.088^‡^

^‡^*χ*2 test, ^¶^Student’s *t* test

Table 5 showed effect size of endometrial preparation on live birth rate in prespecified and exploratory subgroups in each subgroup. No significant interactions were found in any of the subgroups.

**Table 5:** Effect size of endometrial preparation on live birth rate in prespecified and exploratory subgroups in each subgroup

Characteristic	No. of participants	OR (95%CI) ^a^	*P*-value^‡^	*P* for interaction
Endometrial thickness on the day of progesterone administration (mm)				0.952
Tertile 1 (< 9.00)	531	1.11 (0.74, 1.67)	0.609	
Tertile 2 (9.00–10.30)	537	1.22 (0.85, 1.75)	0.292	
Tertile 3 (> 10.30)	570	1.16 (0.82, 1.64)	0.404	
Triple-line endometrial pattern				
A	178	1.02 (0.56, 1.86)	0.956	0.169
B	1344	1.19 (0.94, 1.50)	0.156	
C	116	2.34 (1.04, 5.29)	0.041	
Number of transferred embryos				
1	431	0.94 (0.62, 1.41)	0.760	0.227
2	1192	1.34 (1.05, 1.73)	0.021	
3	15	1.25 (0.16, 9.92)	0.833	
Female age at embryo transfer				
Tertile 1 (< 27)	428	1.15 (0.74, 1.79)	0.529	0.311
Tertile 2 (27–29)	591	1.00 (0.71, 1.42)	0.978	
Tertile 3 (> 29)	619	1.51 (1.08, 2.13)	0.017	
Fertilization type				
IVF	1325	1.17 (0.92, 1.48)	0.200	0.257
ICSI	253	1.25 (0.74, 2.12)	0.410	
IVF + ICSI	60	3.24 (1.04, 10.10)	0.043	
Protocol in the fresh cycle				
Agonist	1489	1.22 (0.98, 1.53)	0.074	0.849
Antagonist	145	1.02 (0.49, 2.11)	0.959	
Other	4			

^‡^*χ*2 test, ^¶^Student’s *t* test

^a^ Adjusted for AFC, BMI, infertility duration, infertility type and cause of infertility except the subgroup variable

The multiple logistic regression model showed no difference between endometrial preparation and live birth while adjusting for potential confounders presented in Table 6. No difference was found in live birth between GnRHa +HRT and HRT before adjusting for covariates (crude OR 1.22, 95%CI, 0.99–1.51, *p* = 0.062) and after PSM (OR 1.47, 95%CI, 0.99–2.83, *p* = 0.068). In addition, there is a marginally difference after adjusting for covariates (aOR 1.56, 95%CI, 1.001–2.41, *p* = 0.048), this finding with *p*-value close to 0.05 represent insufficient empirical evidence. The result of PSM of higher standard of evidence supported ineffectiveness of pretreatment with GnRHa.

**Table 6:** Relationship between endometrial preparation and live birth in different models

Outcome	Crude Model ^a^		Adjusted Model ^b^		PSM-Model ^c^	
	OR (95%CI)	*P*-value^‡^	OR (95%CI)	*P*-value^‡^	OR (95%CI)	*P*-value^‡^
Endometrial preparation						
HRT	Reference		Reference		Reference	
GnRHa+HRT	1.22 (0.99, 1.51)	0.062	1.56 (1.001, 2.41)	0.048	1.47 (0.99, 2.83)	0.068

^‡^*χ*2 test, ^¶^Student’s *t* test

^a^ We did not adjust other covariates in entire cohort

^b^ Adjusted for female age at embryo transfer, female age at oocyte retrieval, infertility duration, infertility type, cause of infertility, AFC, BMI, endometrial thickness, duration of endometrial preparation, triple-line endometrial pattern, protocol in the fresh cycle, gonadotropin duration, gonadotropin dosage, number of oocyte retrieved, number of transferred embryos, number of the available embryos, number of good quality embryos transferred, embryo stage and fertilization type in entire cohort

^C^ Matched cohort

## Discussion

The most advantageous protocol for FET has been the subject of several retrospective studies [12, 13]. To the best of our knowledge, we present the largest analysis to date that compares pretreatment with and without GnRHa with hormone protocol for PCOS patients.

Despite the different experiences and doctors’ preferences with endometrial preparation, there is a lack of evidence to support the superiority of one method over the other. In the past, the most popular protocol of endometrial preparation was HRT, to avoid spontaneous ovulation. A transfer in the HRT cycle is more applicable in women who are not menstruating regularly such as those with ovulation disorder or PCOS and in cases where a better control or a flexible transfer is indicated [14]. HRT cycles can be used with or without a GnRHa for pituitary suppression. There are a few numbers of studies comparing HRT and GnRHa + HRT cycles, however, the results have been conflicting. A prospective randomized study indicated HRT resulted in a similar success rate and lower cost than GnRHa + HRT [14]. However, the sample size was small (*n* = 106 cycles) and did not adjust the confounding covariate. Another randomized controlled trial showed no difference between HRT and GnRHa + HRT in patients with repeated implantation failure (*n* = 67) [15]. One retrospective study compared clinical outcome between GnRH downregulation and HRT, however, they only reported chemical pregnancy and clinical pregnancy, without reporting live birth rate (*n* = 95) [16]. Studies have shown long-term GnRHa before IVF treatment in infertile women with endometriosis increases pregnancy rate [17, 18]. One retrospective study involving 339 cycles of FET patients with adenomyosis and found long-term GnRHa pretreatment significantly improved pregnancy outcomes [19]. However, the beneficial effect of GnRHa in PCOS patients before FET is not known.

Infertile women with PCOS are at a greater risk of early pregnancy loss, aberrant uterine receptivity, and high body mass index [20–22]. When the pituitary is suppressed by the administration of GnRHa, initial follicular activity is inhibited. HRT cycles in the absence of GnRHa suppression can result in a rise in LH, ultimately negatively altering the receptive window of implantation [23]. It can be speculated that GnRHa pretreatment may produce a window of time with improved implantation and increased endometrial receptivity [16, 24, 25]. An additional advantage of GnRHa + HRT is the convenience of better control over the cycle and transfer date [9]. One retrospective study showed that GnRHa pretreatment significantly increased the ongoing pregnancy rate in PCOS women [26]. Another possible mechanism underlying the benefits of GnRHa pretreatment for PCOS is suppression of LH level, E_2_ level, hyperandrogenic level, and GnRH-HCG axis function with inhibition of endometrial inflammation and enhanced expression of endometrial adhesion molecules [27]. GnRHa pretreatment in PCOS patients could lead to an androgen deprivation status, in addition, subsequent HRT cycles for endometrial preparation can synchronize the development of the embryo with the endometrium. However, GnRHa pretreatment has some major disadvantages: the duration of treatment is prolonged, the women may suffer from menopausal symptoms resulting from a hypo-estrogenic state caused by the agonist, and an agonist may lead to ovarian cyst formation.

### Strengths and limitations

The main strengths of our study include the population size, the ability to perform sensitivity analysis with an adequate number of patients and adjusting more confounding covariates. The weaknesses of our study include its retrospective design and the inherent biases therein. Although we used logistic regression to adjust for confounders’ effect on the outcome, it is impossible to control all the confounders.

## Conclusions

Our results suggest that GnRHa + HRT protocols have a comparable chance of live birth to the HRT cycle. Well-designed, prospective clinical trials are needed to further assess the beneficial effect of GnRHa pretreatment of FET on PCOS patients. These studies will aid in patient counseling and in personalizing frozen-thawed embryo transfer.

## Data Availability

The datasets used and/or analyzed during the current study are available from the corresponding author on reasonable request.

## Abbreviations

PCOS: Polycystic ovary syndrome
FET: Frozen-thawed embryo transfer
GnRHa: Gonadotropin-releasing hormone agonist
PSM: Propensity score matching
HRT: Hormone replacement treatment
LH: Luteinizing hormone
FSH: Follicle-stimulating hormone
OHSS: Ovarian hyperstimulation syndrome
PGT: Preimplantation genetic testing
OR: Odds ratio
VIF: Variance inflation factor
CI: Confidence interval
AFC: Antral follicle count
BMI: Body mass index
